# When Should Physicians Consider Referring Elderly Patients with Suspected PFO-Related Stroke for Device Closure?

**DOI:** 10.3390/jcm15010294

**Published:** 2025-12-30

**Authors:** Alisha Varia, David Roberts

**Affiliations:** School of Medicine, University of Liverpool, Liverpool L69 3GE, UK; d.h.roberts@liverpool.ac.uk

**Keywords:** PFO closure, cryptogenic stroke, elderly

## Abstract

**Background**: Guidelines recommend patent foramen ovale (PFO) closure for secondary prevention after cryptogenic stroke in patients aged 18–65 years, but there is limited evidence to guide management of elderly adults. This research aims to assess the efficacy, safety and methodological quality of trials comparing secondary prevention PFO closure with medial therapy alone (MTA) in patients aged ≥ 60 years. **Methods**: A PubMed search identified four studies comparing PFO closure with MTA in elderly patients—PFOSK (South Korea), PT (Taiwan), DEFENSE (South Korea) and PFOG (Germany). Primary analyses evaluated study quality—patient selection, allocation, crossover and adherence. Secondary analyses compared recurrent cerebral ischaemia, mortality, new-onset atrial fibrillation (AF) and disability. **Results**: In 644 patients ≥ 60 years old, PFO closure was associated with a 45% (95% CI 0.35–0.86, *p* = 0.0091) reduction in recurrent cerebral ischaemia and an 85% (95% CI 0.05–0.49, *p* = 0.0016) reduction in mortality. Lower disability scores and increased incidence of new-onset AF (RR 2.15, 95% CI 1.07–4.32, *p* = 0.0306) was observed in closure groups. Study quality was limited by heterogeneity in medical regimens and closure protocols, crossover between treatment arms and imbalances in baseline characteristics, with closure groups generally younger and possessing larger shunt sizes. **Conclusions**: In patients aged ≥ 60 years, PFO closure appears to reduce the risk of the recurrence of ischaemic events and mortality, particularly in those with ‘high-risk’ PFO features. However, variability in study designs and low event rates limit certainty. Large, standardised trials are warranted to provide evidence for guideline recommendations in this population.

## 1. Introduction

ESC guidelines [1] recommend patent foramen ovale (PFO) closure as secondary prevention in patients aged 18 to 65 years with a cryptogenic stroke. They suggest case-by-case decision-making when patients over 65 present with this condition, providing no single recommendation for treatment [1,2]. NHS England [2] only commissions PFO closure as a secondary prevention in patients aged 60 years and under. Probability of causation has traditionally been determined using the paradoxical embolism (RoPE) score, with scores of seven and above indicating a greater likelihood of PFO causation for a cryptogenic stroke [3].

PFO presence ranges from 16 to 18% in patients above 55 years with a cryptogenic stroke [4], three times more prevalent compared to those patients with stroke of known cause [5]. In a systematic review and meta-analysis by Mazzuco et al., planned stratification by age showed a 150% increased risk of recurrent stroke in the presence of PFO in patients over 65 [6]. Despite this, there remains limited trial evidence exploring the benefits of PFO closure in elderly patients suffering a cryptogenic stroke.

This review aims to evaluate evidence for PFO closure in adults aged ≥ 60 years who experience PFO-related cerebrovascular events, specifically compared to those who receive secondary prevention with antiplatelet or anticoagulation therapy alone.

## 2. Materials and Methods

### 2.1. Literature Search and Paper Selection

**Figure 1:**
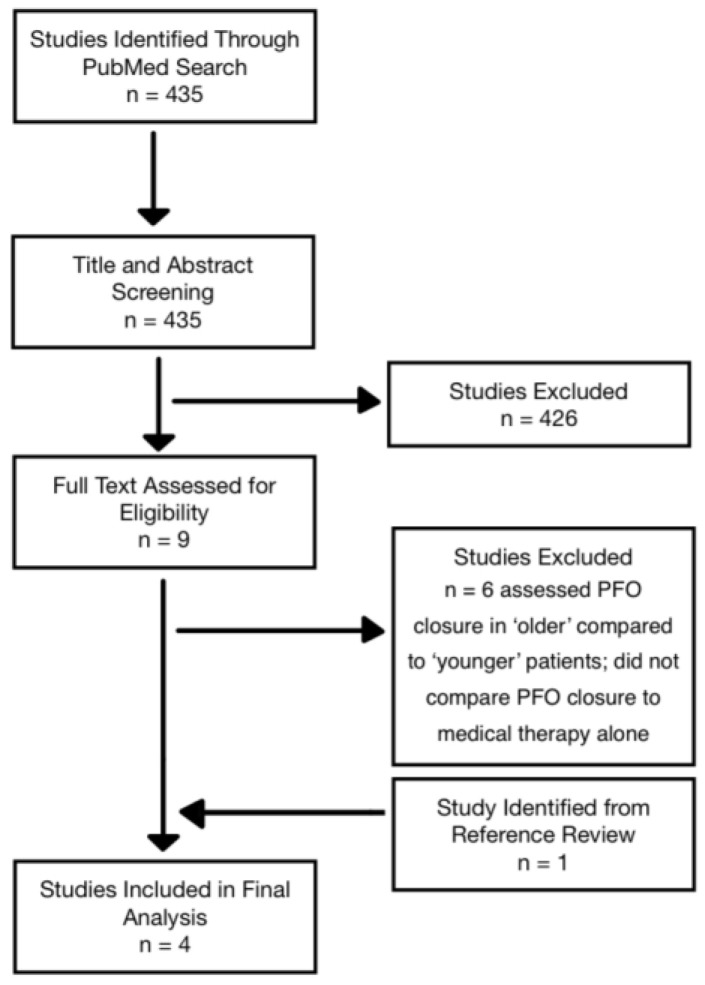
PRISMA flow diagram of study selection.

This review was conducted as a structured narrative review incorporating systematic elements. Because evidence for PFO closure in adults ≥60 years is sparse, we intentionally adopted a broad search strategy to maximise sensitivity. The predefined search terms (Table 1) produced 435 results, of which 426 were excluded during title and abstract screening due to irrelevance arising from this broad approach (Figure 1). Additional six studies were excluded because they compared elderly versus younger closure populations rather than closure versus medical therapy alone. Screening of study references yielded an additional paper, comprising age-based subgroup analyses of a previous randomised controlled trial (RCT). Four studies met final inclusion criteria. A PRISMA flow diagram was used to transparently document the selection process.

### 2.2. Analysis

Primary analysis includes the methodological quality of included studies, assessed according to predefined criteria—patient selection, treatment allocation, crossover between treatment groups and adherence to assigned treatment. Secondary analysis pooled available event data for recurrent stroke and TIA, new-onset atrial fibrillation (AF), peri-operative complications and death. Subgroup analyses then determine any stratification factors associated with benefits from PFO closure in elderly patients. Measurements of disability, such as modified Rankin Scale (mRS), are reported.

Statistical analyses on pooled event rates were obtained from Wald Z-tests applied to Mantel–Haenszel-derived pooled relative risk (RR).

## 3. Results

### 3.1. Quality of Included Studies

Trials without given names were referred to as PFO(x), with “x” representing the country of conduct. The four studies included are PFOSK (South Korea—Lee at al., 2024) [7], PFOT (Taiwan—Chen et al., 2023) [8], DEFENSE (Kwon et al., 2021) [9] and PFOG (Germany—Poli et al., 2021) [10]. These studies compared the efficacy of PFO closure, followed by at least three months of dual antiplatelet therapy (DAPT), with standard medical therapy alone (MTA), which included antiplatelet agents and/or anticoagulation therapy. PFO was diagnosed by echocardiography and bubble study in all studies except PFOT, which used a transcranial Doppler. The Cardia PFO occluder was used in PFOT and Amplatzer PFO occluder in DEFENSE and PFOG (Table 2 and Table A1). A range of devices including Amplatzer, Cocoon and Figulla Flex II PFO occluders were used in PFOSK.

PFOT and PFOG included all patients over the age of 18, with planned subgroup analyses for patients ≥ 60 or >60 years, respectively. DEFENSE was a post hoc subgroup analysis of patients aged 60 years or older from the original study, while PFOSK specifically included those aged 60 years or older. DEFENSE only included patients with high-risk PFO features, defined as the presence of atrial septal aneurysm (ASA) or hypermobility and/or PFO ≥ 2 mm in diameter visualised on transoesophageal echocardiography. PFOG and PFOSK included planned subgroup analysis of patients with ‘high-risk’ echocardiographic features for thromboembolism.

Primary outcome in PFOT, PFOSK and DEFENSE was a composite of recurrent cerebral ischaemic events (ischaemic stroke (IS) or TIA), with additional all-cause mortality in PFOT. Primary outcome in PFOG was recurrent IS and intracranial haemorrhage. Cerebral ischaemia was assessed using magnetic resonance imaging (MRI) and angiography (MRA) or computed tomography angiography (CTA).

PFOSK and PFOT performed patient allocation using patient-centred multidisciplinary consensus and shared decision-making with the patient and family (Table 3). DEFENSE performed randomised 1:1 allocation, and PFOG used a predefined standard operating procedure (SOP) with an age-cutoff of 70 years for closure of a PFO with ‘high-risk’ features.

Adherence to MTA or percutaneous closure was only reported in PFOG, where as-treated analyses were performed. A total of 28 and 17 patients from closure and MTA groups, respectively, crossed over, either through patient- or physician decision. PFOSK reported 2.2% of MTA patients were taking no antithrombotic therapy at 30 days after stroke.

PFOG and PFOSK reported patients in the MTA groups were significantly older than those in PFO closure groups at baseline. Both described a greater prevalence of vascular risk factors—hypertension (HTN), smoking history, hyperlipidaemia, obesity, diabetes, coronary artery disease (CAD) or prior myocardial infarction (MI), chronic kidney disease (CKD), prior deep vein thrombosis (DVT) or pulmonary embolism (PE), migraine or cancer history—within MTA groups, although none are significantly different. DEFENSE reported no significant differences between baseline characteristics of closure and MTA groups.

PFOSK and PFOT reported ‘high-risk’ PFO features for thromboembolism. In PFOSK, there was a low prevalence of large shunt size in MTA group, while 22% and 70% of the closure group also did not have a large shunt or ASA, respectively. The PFOT MTA group had a greater prevalence of small shunts (63% vs. 20% in the closure group).

Baseline median mRS was not significantly different between the closure group and MTA (3 vs. 2, respectively, *p* = 0.989) in PFOT; meanwhile, in PFOG, ‘high-risk’ closure group had a similar number of patients with a baseline mRS score of 0 compared to ‘high-risk’ MTA (88% vs. 89%, respectively).

### 3.2. Secondary Outcomes

A total of 966 patients were included in the four studies, 644 ≥60 years old, of which 392 were assigned to MTA and 252 to the PFO closure arm.

Collated incidence of recurrent cerebral ischaemia in all four studies was 9.1% in closure arm (*n* = 23) and 15.6% in MTA (*n* = 65)—this includes all-cause mortality data from PFOT due to sole reporting of composite primary outcome event rates. This corresponds to a significant reduction in recurrent cerebral ischaemia following PFO closure, with an RR of 0.55 (95% CI 0.35–0.86, *p* = 0.0091). Mortality rate, combining all studies except PFOT, to prevent duplicate use of results, was 1.1% following PFO closure (*n* = 3) and 7.9% in the MTA (*n* = 31), RR 0.15 (95% CI 0.05–0.49, *p* = 0.0016) in patients over 60 years.

New-onset AF occurred in 18 patients following PFO closure and in 13 patients in the MTA (RR 2.15, 95% CI 1.07–4.32, *p* = 0.0306). A total of 15 and 13 events with closure and MTA, respectively, were from PFOSK, which was the only paper to report a significant difference in the 5-year event rate between groups (*p* = 0.026). However, PFOT found no significant difference in new-onset AF between elderly and younger patient groups. DEFENSE only reported two cases of AF and did not specify the age or treatment arm. Modified Rankin scale (mRS) score at follow-up was reduced following PFO closure, i.e., better outcomes, compared to MTA in PFOT (1 (IQR 0–2) vs. 1 (IQR 0–3); *p* = 0.002) and PFOG (mRS score of 0/1—77% in high-risk closure group vs. 68% in high-risk MTA; RR 0.72).

Subgroup analyses identified that elderly patients with high-risk PFO features (aneurysmal septum, large shunt) benefit mostly from PFO closure. In PFOT, a significant reduction in ischaemic stroke risk was found with closure (HR 0.36, 95% CI 0.18–0.75, *p* = 0.006), compared to the overall cohort (HR 0.52, *p* = 0.034). PFOSK also showed a significant reduction in ischaemic stroke with PFO closure in propensity-score matched, high-risk patients (HR 0.47, 95% CI 0.23–0.95, *p* = 0.035), compared to a non-significant risk decrease in the overall cohort (HR 0.58, *p* = 0.107). DEFENSE showed a high incidence of TIA/stroke in patients over 70 years (HR 11.64, *p* = 0.03) and these benefited the most from PFO closure, but this finding was limited by a large confidence interval due to a small sample size.

## 4. Discussion

### 4.1. Summary of Evidence

We report a dual analysis as follows: examination of trial evidence quality and comparison of PFO closure versus medical management in patients over 60 years of age. This approach was driven by the limited studies focusing solely on elderly patients and the lack of guideline recommendations for the management of these patients.

The collated findings provide evidence that PFO closure in elderly patients decreases the incidence of recurrent IS and TIA and reduces mortality and disability scores compared to medical therapy alone. However, this evidence is limited by the comparability of trial protocols and results, including variations in treatment allocation and patient characteristics, medical therapies and closure devices, result analysis and treatment adherence.

Subgroup analyses in PFOSK and DEFENSE show that elderly patients with high-risk PFO features—ASA or large shunt size (≥2 mm on TEE)—may have the greatest benefit from closure. The probability of incidental PFO finding following cryptogenic stroke is 48% in those over 55, compared to just 20% in younger patients, both of which reduced to 26% and 9%, respectively, when a concomitant ASA was detected [4].

### 4.2. Comparison with Current Guideline Recommendations

Current ESC guidelines [1] strongly recommend PFO closure as secondary prevention for cryptogenic stroke in adults aged 18–65 years when a high probability of PFO causality is established. For individuals > 65 years, the guidelines advise case-by-case decision-making, acknowledging the absence of robust evidence in this age group accounting for personal confounders of surgical or medical intervention [1]. NHS England policy [2] similarly restricts percutaneous PFO closure to patients ≤ 60 years, reflecting comparable concerns regarding diagnostic certainty and increasing atherosclerotic risks in elderly populations.

Both ESC and NHS guidance [1,2] highlight the use of the RoPE score to estimate the likelihood of a PFO-related stroke but also note its limitations—particularly the lack of external validation. Although the guidelines acknowledge that other tools may complement RoPE scoring, they do not specify alternatives, such as the PASCAL classification system.

The evidence evaluated in this review suggests that selected elderly adults may benefit from percutaneous PFO closure, consistent with findings in younger populations. These findings reinforce the need for rigorous trials in those over 60 years of age to inform guideline updates. Until such data are available, incorporating anatomical high-risk features may assist in defining causal attribution of cerebral ischaemia and support personalised decision-making in elderly patients.

### 4.3. Strengths

This review uniquely emphasises methodological quality as its primary objective. By combining structured selection criteria, transparent reporting and pooled outcome assessment, it provides a unified appraisal of the available evidence base and highlights methodological issues that impede clinical translation.

### 4.4. Limitations of Trial Evidence

The interpretation of trial evidence in elderly adults remains constrained by several methodological and practical issues. Firstly, medical therapies and closure devices and procedures were not standardised across or within studies, with heterogeneity in anti-thrombotic regimes, closure devices and post-operative management. This variability limits comparability and introduces potential bias in outcome interpretation.

Secondly, adherence of assigned treatment was inconsistent—for example, forty-five patients in PFOG crossed over between treatment arms, while 2.2% of MTA patients in PFOSK were not on any antithrombotic therapy at 30 days. Those that crossed over tended to be younger and possess fewer risk factors, yet had larger shunts, presenting a selection bias that paradoxically produced a non-significant higher recurrence rate with closure. Furthermore, treatment allocation in PFOG was determined by a procedural age cut-off of 70 years, with analyses performed on a 60-year threshold, further complicating interpretation.

Thirdly, marked differences in baseline characteristics between treatment arms were consistently observed. Patients in MTA groups were generally elderly, with greater prevalence of vascular comorbidities such as HTN, diabetes and CAD, whereas those in closure groups often had larger shunt sizes and ASA—features associated with higher embolic risks. These imbalances confound results as vascular risk factors of PFO-independent determinants of recurrent stroke.

Event rates across studies were generally low, resulting in wide confidence intervals and limited statistical power to detect meaningful differences. Mortality outcomes were inconsistently reported, precluding firm attribution to PFO closure, medical therapy or recurrent stroke. Importantly, due to limited available data, pooled analyses necessarily combined intention-to-treat and as-treated, which introduces bias. This reflects deficiencies in reporting rather than analytic choice and highlights the need for standardised methodologies in future research.

The observed two-fold increase in new-onset AF following PFO closure may partially reflect greater baseline risk in elderly patients with pre-existing cardiovascular disease.

Collectively, these limitations highlight the need for adequately powered RCTs specifically designed for patients over 60 years with standardised endpoint definitions, device and medical therapy protocols and stratification by high-risk PFO features.

## 5. Recommendations and Clinical Implications

**Figure 2:**
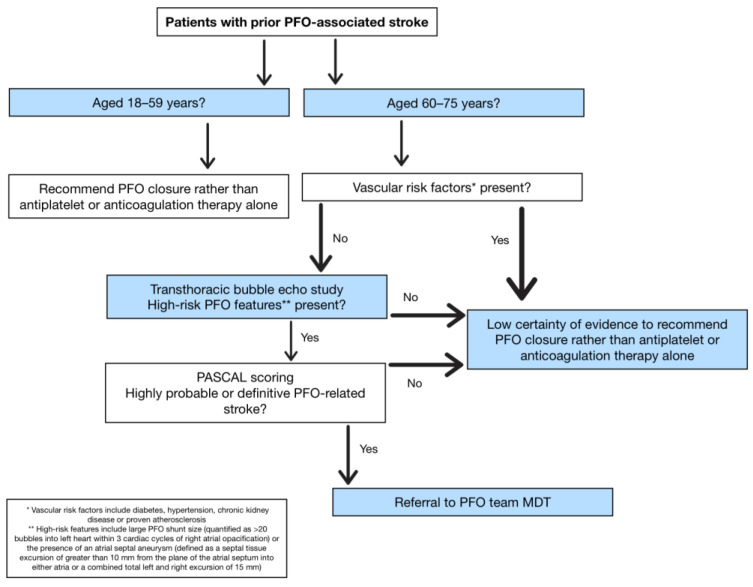
Example algorithm for patent foramen ovale (PFO) closure decision-making.

The findings in this review support the consideration of patients aged 60–75 years presenting with a cryptogenic stroke without vascular risk factors (diabetes, HTN, CKD) or proven atherosclerosis for PFO closure (Figure 2). Transthoracic bubble echo study should focus on identifying large PFO shunt size (quantified as >20 bubbles into left heart within three cardiac cycles of right atrial opacification [11]) and the presence of an ASA (defined as a septal tissue excursion of greater than 10 mm from the plane of the atrial septum into either atria or a combined total left and right excursion of 15 mm [12]).

Elderly patients inadvertently have a lower RoPE score due to age and comorbidity scoring, highlighting its limitation to determine eligibility for PFO closure. PASCAL is an alternative assessment tool that combines RoPE scores with the presence of high-risk PFO features [13]. Meta-analysis showed a greater reduction in risk of recurrent ischaemic stroke when using PASCAL categorisation to select patients for PFO closure age 18–60 years [13]. We suggest PASCAL classification (Table 4) for determining causal attribution of recurrent cerebral ischaemia and selection of elderly patients for potential PFO closure, aligned with current guidelines. Those patients scored with highly probable or definitive outcome of PFO-related stroke may be considered for PFO closure by referral to the PFO team MDT. All decision-making should also consider patient preference and weighing individual risk-to-benefit analysis.

Future studies of PFO closure in patients > 60 years of age must include adequately powered RCTs with:

- standardised medical and closure protocols
- predefined upper age limits
- consistent ITT reporting
- stratification by high-risk PFO anatomy
- robust follow-up and adherence reporting

Such trials are essential to generate definitive recommendations for patients ≥ 60 years.

## 6. Conclusions

Evidence from the four studies evaluating PFO closure in patients aged ≥ 60 years demonstrates a consistent trend towards reduced recurrent cerebral ischaemia, lower mortality and improved functional outcomes compared with MTA. However, interpretation must account for substantial heterogeneity between studies. While pooled analyses suggest meaningful benefit, differences in closure devices, medical regimens, participant allocation and baseline comorbidities—combined with low event rates—introduce uncertainty and limit generalizability.

Importantly, the greatest benefit appears concentrated in those with ‘high-risk’ PFO features—large shunt size and ASA—supporting the use of detailed assessment tools such as PASCAL to evaluate causality.

Taken together, the existing evidence supports MDT referral for selected elderly individuals, but it remains insufficient to form definitive guideline recommendations.

## Figures and Tables

**Table 1:** Search terms.

Search Terms
(Patent foramen ovale) OR (PFO) OR (foramen ovale)
AND (Closure) OR (Occlu *) OR (Surg *)
AND (High risk) OR (Elderly) OR (Older) OR (Old)
AND case reports OR clinical study OR clinical trial OR observational trial OR randomised controlled trial
AND English language
AND year 2015:2025
AND humans

* indicates the truncation symbol to identify multiple word endings from the provided root.

**Table 2:** Study protocols assessing medical management vs. patent foramen ovale (PFO) closure in patients over 60 years.

	Poli et al., 2021 [10]	Kwon et al., 2021 [9]	Chen et al., 2023 [8]	Lee et al., 2024 [7]
**Name**	PFOG	DEFENSE	PFOT	PFOSK
**Place**	Germany	South Korea	Taiwan	South Korea
**Type of Study**	Prospective case series	Randomised controlled trial	Prospective cohort	Retrospective cohort
**Aim**	Compare interventional and medical PFO-management in cryptogenic IS/TIA patients, including patients > 60 years	Investigate the benefit of PFO closure in elderly adults	Investigate the efficacy and safety of PFO closure in non-elderly and elderly patients	Assess whether PFO closure is also beneficial in elderly patients
**Inclusion Criteria**	Acute IS or TIA and PFO diagnosis High-risk PFO ^1^	IS within the previous six months and a high-risk PFO with no other identifiable causes	Cryptogenic IS or cryptogenic TIA, PFO and aged over 18 years	Aged ≥ 60 years with cryptogenic IS diagnosed with PFO
**PFO Closure Device and Medical Management**	Amplatzer PFO-occluder 25 or 35 mm Performed after median 54 days after index TIA or IS DAPT aspirin and clopidogrel regime after closure SAPT after 3 or 6 months once residual shunt or thrombi excluded	Amplatzer PFO Occluder Recommended DAPT (aspirin 100 mg/day with clopidogrel 75 mg/day) for at least 6 months after closure	Cardia PFO Occluder Recommended antiplatelet regimes included aspirin (100 mg once a day) or clopidogrel (75 mg once a day) alone and DAPT. Oral anticoagulant (warfarin or non-vitamin K antagonist oral anticoagulant) would be administered to patients diagnosed with periprocedural AF	Amplatzer PFO Occluder, Cocoon PFO Occluder and Figulla Flex II PFO Occluder Recommended for DAPT regimen (aspirin 100 mg/day with clopidogrel 75 mg/day) for at least 6 months after closure
**Medical Therapy Alone (MTA)**	SAPT or oral anticoagulation preferably with DOAC	Antiplatelet or anticoagulation chosen by local investigator Antiplatelet therapy included aspirin, aspirin with clopidogrel 75 mg/day or aspirin with cilostazol 200 mg/day Warfarin was used to maintain the target international normalised ratio of 2.0 to 3.0	As medical regime above	Antiplatelet or anticoagulation therapy closed by attending physician
**Patient Allocation**	According to institution SOP—age cut off of 70 years old for interventional management of high-risk PFO; individual clinical considerations	Randomised 1:1	Eligible for PFO closure unless there was active bleeding, allergy to radiographic contrast, acute pulmonary oedema or active systemic infection. Multidisciplinary assessment of potential benefits and risks of PFO closure with patient-shared decision-making	Decision to choose between PFO closure or medical therapy for an individual was made through consensus, accounting for the interpretation of neurological and cardiac imaging, the possibility of other sources of cardiac embolism, the presence of comorbidities, assessment of PFO morphology and the procedural risk of PFO closure at each centre

^1^ high risk defined as the presence of an ASA, spontaneous or high-grade right to left shunt during valsalva manoeuvre. IS—ischaemic stroke; TIA—transient ischaemic attack; PFO—patent foramen ovale; DOAC—direct oral anticoagulant; SAPT—single antiplatelet therapy; DAPT—dual antiplatelet therapy; SOP—standard operating procedure; ASA—atrial septal aneurysm,

**Table 3:** Study results assessing medical management vs. patent foramen ovale (PFO) closure in patients over 60 years.

	Poli et al., 2021 [10]	Kwon et al., 2021 [9]	Chen et al., 2023 [8]	Lee et al., 2024 [7]
**Name**	PFOG	DEFENSE	PFOT	PFOSK
**Sample size (intention-to-treat)**	All patient within study Closure 157 MTA 37	≥60 years old Closure 13 MTA 21	≥60 years old Closure 35 MTA 43	≥60 years old Closure 161 MTA 276
**Baseline characteristic differences between groups**	When comparing high-risk PFO ^2^ in >60 years, closure median age 66, MTA median age 75 MTA group had higher rates of diabetes, smoking history Vascular risk factors more common in MTA group	No significant differences	Significantly greater number had small shunts in MTA group 62.8% vs. 20% in closure	MTA group had older median age 96.2 years vs. 66.2 years in closure group Greater stroke risk factors in MTA group—not individually significantly different but may be different when combined MTA group had lower frequency of large shunt size 51.8% vs. 88.2% in closure Within closure group, 19 did not have large shunts
**Adherence to treatment**	28 from closure had MTA (17 on patient decision, 11 by physician recommendation) 17 from MTA had closure (7 on patient decision, 10 by physician recommendation)	7 in initial parent study declined closure—no data on age subgroup analyses	N/A ^1^	In MTA group, 2.2% had no antithrombotic therapy at 30 days after stroke
**Follow-up period**	Mean 2.8 years	Median 4.4 years closure group Median 2.5 years MTA group	Mean 2.5 years	Median 3.9 years
**Reported results of ischaemic events**	As-treated analysis 146 underwent closure 48 had MTA Comparing high-risk PFO in >60 years 43 closure vs. 28 MTA IS recurrence 7% closure vs. 4% MTA RR 1.95 0.21–17.85 All other events—not IS, TIA, systemic embolism or PFO related death—7% closure, 18% MTA RR 0.49 0.12–2.02	Intention-to-treat analysis DAPT—most common in MTA SAPT most used after closure Difference in recurrent IS or TIA—2-year event rate, 24.6%; HR, 7.36; 95% CI, 0.28 to 195.81; log-rank *p* = 0.07 When compared for those over 70—higher difference 2-year event rate, 80%; HR, 11.64; 95% CI, 0.43 to 318.81; log-rank *p* = 0.03 4 events in MTA group over 60—3 IS, 1 TIA 0 in closure	Recurrent TIA, IS and all-cause mortality was 8.6% closure vs. 23.3% MTA, approaching statistical significance, HR 0.26 0.07–1.01 *p* = 0.051 PFO closure had a numerically higher probability of a favourable outcome at 180 days in the elderly (OR 2.09, 95% CI 0.76–6.25, *p*= 0.185), but not significant	Most common at 30 days after stroke was antiplatelet alone for both groups Risk of IS or TIA was significantly lower in the PFO closure group HR 0.52; 95% CI 0.29–0.95; *p* = 0.034 Rates of IS outcomes were more prominent in patients with a high-risk PFO ^3^; the PFO closure group showed significantly lower risks of recurrent IS (HR: 0.36; 95% CI: 0.18–0.75; *p* = 0.006) and the composite outcome of IS or TIA (HR: 0.36; 95% CI: 0.19–0.69; *p* = 0.002) Risk of death (HR 0.19; 95% CI 0.04–0.81; *p* = 0.025) and the composite outcome of IS, TIA or systemic embolisation (HR 0.55; 95% CI 0.31–0.99; *p* = 0.046) were significantly lower in the PFO closure group

^1^ N/A—data not reported within study. ^2^ high risk defined as the presence of an ASA, spontaneous or high-grade right to left shunt during valsalva manoeuvre. ^3^ high risk defined as the presence of an ASA, hypermobility or size of ≥2 mm on transoesophageal echocardiography. IS—ischaemic stroke; TIA—transient ischaemic attack; PFO—patent foramen ovale; SAPT—single antiplatelet therapy; DAPT—dual antiplatelet therapy; ASA—atrial septal aneurysm; RR—relative risk; HR—hazard ratio.

**Table 4:** Risk of paradoxical embolism (RoPE) and PFO-associated stroke causal likelihood (PASCAL) scoring systems.

**RoPE Score**		
**Age (years)**		**Points**
18–29		5
30–39		4
40–49		3
50–59		2
60–69		1
≥70		0
Characteristic		
No history of hypertension		1
No history of diabetes		1
No history of stroke/transient ischaemic attack		1
Non-smoker		1
Cortical infarct on imaging		1
Total score ≥ 7—stroke likely to be cause by PFO (≥72% probability)		
**PASCAL Score**		
**High-risk PFO features**	**RoPE score > 7**	**PFO-related stroke**
Straddling thrombus	Yes	Definitive
	No	Definitive
ASA or large shunt size AND PE or DVT preceding index infarct	Yes	Highly probable
	No	Probable
ASA AND/OR large shunt	Yes	Probable
	No	Possible
Small shunt with no ASA	Yes	Probable
	No	Unlikely

PFO—patent foramen ovale; ASA—atrial septal aneurysm; PE—pulmonary embolism; DVT—deep vein thrombosis.

## Data Availability

No new data were created for this research.

## Abbreviations

The following abbreviations are used in this manuscript:

ESC	European Society of Cardiology
PFO	Patent foramen ovale
NHS	National Health Service
RoPE	Risk of paradoxical embolism
RCT	Randomised controlled trial
TIA	Transient ischaemic attack
AF	Atrial fibrillation
mRS	Modified Rankin scale
RR	Relative risk
MTA	Medical therapy alone
ASA	Atrial septum aneurysm
IS	Ischaemic stroke
MRI	Magnetic resonance imaging
MRA	Magnetic resonance angiography
CTA	Computed tomography angiography
SOP	Standard operating procedure
HTN	Hypertension
CAD	Coronary artery disease
MI	Myocardial infarction
CKD	Chronic kidney disease
DVT	Deep vein thrombosis
PE	Pulmonary embolism
CI	Confidence interval
IQR	Interquartile range
HR	Hazard ratio
PASCAL	PFO-associated stroke causal likelihood

## Appendix A

**Table A1:** Studies assessing medical management vs. patent foramen ovale (PFO) closure in patients over 60 years.

	Poli et al., 2021 [10]	Kwon et al., 2021 [9]	Chen et al., 2023 [8]	Lee et al., 2024 [7]
**Name**	PFOG	DEFENSE	PFOT	PFOSK
**Place**	Germany	South Korea	Taiwan	South Korea
**Type of study**	Prospective case series	Randomised controlled trial	Prospective cohort	Retrospective cohort
**Aim**	Compare interventional and medical PFO management in cryptogenic IS/TIA patients, including patients > 60 years	Investigate the benefit of PFO closure in elderly adults	Investigate the efficacy and safety of PFO closure in non-elderly and elderly patients	Assess whether PFO closure is also beneficial in elderly patients
**Recruiting period**	Consecutive patients between March 2012 and September 2016	September 2011–October 2017	January 2013–October 2021	Consecutive patients between January 2008 and December 2020
**Inclusion criteria**	Acute IS or TIA and PFO diagnosis High-risk PFO ^2^	IS within the previous six months and a high-risk PFO with no other identifiable causes	Cryptogenic IS or cryptogenic TIA, PFO and aged over 18 years	Aged ≥60 years with cryptogenic IS diagnosed with PFO
**Exclusion criteria**	N/A ^1^	Significant (≥50%) cerebral artery steno-occlusion or lacunar infarcts	Diagnosed with pulmonary arteriovenous malformation according to the transcatheter procedure Follow-up period of <6 months	Other identifiable mechanisms of stroke Large-artery disease (≥50% steno-occlusion in the intracranial or extracranial arteries) Significant atherothrombosis (plaque thickness of ≥4 mm) in the thoracic aorta Cardiac origin of embolism—presence of cardiac condition with a high embolic risk, such as atrial fibrillation, valvular heart disease (presence of a prosthetic valve or moderate-to-severe rheumatic mitral stenosis), acute myocardial infarction with a mural thrombus, endocarditis or systolic heart failure with an ejection fraction of ≤40% Stroke caused by small vessel disease (defined as a <1.5 cm in diameter, deep vessel) infarction, without evidence of relevant large artery disease or cardiac embolism
**PFO closure device and medical management**	Amplatzer PFO occluder 25 or 35 mm Performed after median 54 days after index TIA or IS DOAC or heparin-bridging therapy DAPT aspirin and clopidogrel regime after closure SAPT after 3 or 6 months once residual shunt or thrombi excluded	Amplatzer PFO occluder Recommended DAPT (aspirin 100 mg/day with clopidogrel 75 mg/day) for at least 6 months after closure	Cardia PFO Occluder Recommended antiplatelet regimes included aspirin (100 mg once daily) or clopidogrel (75 mg once daily) alone and DAPT Oral anticoagulant (warfarin or non-vitamin K antagonist oral anticoagulant) would be administered to patients diagnosed with periprocedural AF	Amplatzer PFO Occluder, Cocoon PFO Occluder and Figulla Flex II PFO Occluder Recommended for DAPT regimen (aspirin 100 mg/day with clopidogrel 75 mg/day) for at least 6 months after closure
**Medical therapy alone (MTA)**	SAPT or oral anticoagulation preferably with DOAC	Antiplatelet or anticoagulation chosen by local investigator Antiplatelet therapy included aspirin, aspirin with clopidogrel 75 mg/day or aspirin with cilostazol 200 mg/day Warfarin was used to maintain the target international normalised ratio of 2.0 to 3.0	Antiplatelet regimes included aspirin (100 mg once daily) or clopidogrel (75 mg once daily) alone and DAPT Oral anticoagulant (warfarin or non-vitamin K antagonist oral anticoagulant) would be administered to patients diagnosed with periprocedural AF	Antiplatelet or anticoagulation therapy closed by attending physician
**Patient allocation**	According to institution SOP—age cut off of 70 years old for interventional management of high risk PFO; individual clinical considerations	Randomised 1:1	Eligible for PFO closure unless there was active bleeding, allergy to radiographic contrast, acute pulmonary oedema or active systemic infection The multidisciplinary stroke team would discuss the potential benefits and risks of PFO closure with the family or patient in a shared decision-making conference	Decision to choose between PFO closure or medical therapy for an individual was made through consensus, accounting for the interpretation of neurological and cardiac imaging, the possibility of other sources of cardiac embolism, the presence of comorbidities, assessment of PFO morphology and the procedural risk of PFO closure at each centre
**Sample size (intention-to-treat)**	All patient within study Closure 157 MTA 37	≥60 years old Closure 13 MTA 21	≥60 years old Closure 35 MTA 43	≥60 years old Closure 161 MTA 276
**Baseline characteristic differences between groups**	When comparing high-risk PFO in >60 years closure median age 66, MTA median age 75 MTA group had higher rates of diabetes, smoking history Vascular risk factors more common in MTA group	No significant differences	Significantly greater number had small shunts in MTA group 62.8% vs. 20% in closure	MTA group had older median age 96.2 years vs. 66.2 years in closure group Greater stroke risk factors in MTA group—not individually significantly different but may be different when combined MTA group had lower frequency of large shunt size 51.8% vs. 88.2% in closure Within closure group, 19 did not have large shunts
**Adherence to treatment**	28 from closure had MTA (17 on patient decision, 11 by physician recommendation) 17 from MTA had closure (7 on patient decision, 10 by physician recommendation)	7 in initial parent study declined closure—no data on age subgroup analyses	N/A ^1^	In MTA group, 2.2% had no antithrombotic therapy at 30 days after stroke
**Characteristics of crossovers**	Larger shunts, less frequent ASA, more commonly primary TIA rather than IS, less frequent diabetes and prior CAD/MI, more frequent HTN and hyperlipidaemia, in patients who crossed over to MTA compared to non-crossovers Younger, larger shunt, fewer ASA, more commonly primary TIA, less frequent hyperlipidaemia, diabetes and CAD or prior MI, in patients who crossed over to closure compared to non-crossovers	N/A ^1^	N/A ^1^	N/A ^1^
**Follow-up period**	Mean 2.8 years	Median 4.4 years closure group Median 2.5 years MTA group	Mean 2.5 years	Median 3.9 years
**Reported results**	As-treated analysis 146 underwent closure 48 had MTA—19 SAPT, 23 DOAC, 6 phenprocoumon Comparing high-risk PFO in >60 years 43 closure vs. 28 MTA IS recurrence 7% closure vs. 4% MTA RR 1.95 0.21–17.85 All other events—not IS, TIA, systemic embolism or PFO related death—7% closure, 18% MTA RR 0.49 0.12–2.02	Intention-to-treat analysis DAPT—most common in MTA SAPT most used after closure Difference in recurrent IS or TIA—2-year event rate, 24.6%; HR, 7.36; 95% CI, 0.28 to 195.81; log-rank *p* = 0.07 When compared for those over 70—higher difference 2-year event rate, 80%; HR, 11.64; 95% CI, 0.43 to 318.81; log-rank *p* = 0.03 4 events in MTA group over 60—3 IS, 1 TIA 0 in closure	Recurrent TIA, IS and all-cause mortality was 8.6% closure vs. 23.3% MTA, approaching statistical significance, HR 0.26 0.07–1.01 *p* = 0.051 PFO closure had a numerically higher probability of a favourable outcome at 180 days in the elderly (OR 2.09, 95% CI 0.76–6.25, *p*= 0.185), but not significant Among the elderly, the PFOC group had a lower median mRS at 180 days than the non-PFOC group (IQR 0–2 vs. 0–3, *p* = 0.002)	Most common at 30 days after stroke was antiplatelet alone for both groups Risk of IS or TIA was significantly lower in the PFO closure group HR 0.52; 95% CI 0.29–0.95; *p* = 0.034 Rates of IS outcomes were more prominent in patients with a high-risk PFO ^3^; the PFO closure group showed significantly lower risks of recurrent IS (HR: 0.36; 95% CI: 0.18–0.75; *p* = 0.006) and the composite outcome of IS or TIA (HR: 0.36; 95% CI: 0.19–0.69; *p* = 0.002) Risk of death (HR 0.19; 95% CI 0.04–0.81; *p* = 0.025) and the composite outcome of IS, TIA or systemic embolisation (HR 0.55; 95% CI 0.31–0.99; *p* = 0.046) were significantly lower in the PFO closure group Rate of AF was higher in the PFO closure group (HR 2.28; 95% CI 1.08–4.82; *p* = 0.030)
**Result**	Closure group had a non-significantly higher risk of recurrence in >60-year-olds	Benefit of closure is greater in those ≥60 years old than in younger patients	No statistically significant difference between groups but some benefit of closure for over ≥60-year-olds Majority of MTA group had small shunt size and may not be eligible for interventional closure	Reduced risk of recurrent IS or TIA in closure group 60 years old, more significant in those with high-risk PFO Differences in baseline characteristics may confound results

^1^ N/A—data not reported within study. ^2^ high risk defined as the presence of an ASA, spontaneous or high-grade right to left shunt during valsalva manoeuvre. ^3^ high risk defined as the presence of an ASA, hypermobility or size of ≥2 mm on transoesophageal echocardiography. IS—ischaemic stroke; TIA—transient ischaemic attack; PFO—patent foramen ovale; DOAC—direct oral anticoagulant; SAPT—single antiplatelet therapy; DAPT—dual antiplatelet therapy; SOP—standard operating procedure; ASA—atrial septal aneurysm, CAD—coronary artery disease; MI—myocardial infraction; HTN—hypertension; RR—relative risk; HR—hazard ratio.
